# Augmented Therapeutic Potential of EC-Synthetic Retinoids in Caco-2 Cancer Cells Using an In Vitro Approach

**DOI:** 10.3390/ijms23169442

**Published:** 2022-08-21

**Authors:** Mohamed R. Abdelaal, Esraa Ibrahim, Mohamed R. Elnagar, Sameh H. Soror, Hesham Haffez

**Affiliations:** 1 Biochemistry and Molecular Biology Department, Faculty of Pharmacy, Helwan University, Cairo 11795, Egypt; 2 Center of Scientific Excellence “Helwan Structural Biology Research, (HSBR)”, Helwan University, Cairo 11795, Egypt; 3 Department of Pharmacology and Toxicology, Faculty of Pharmacy, Al-Azhar University, Cairo 11823, Egypt

**Keywords:** EC-synthetic retinoids, AC261066, CD437, CD2665, ATRA, Caco-2, P-gp1, BCRP, MRP1

## Abstract

Colorectal cancer therapies have produced promising clinical responses, but tumor cells rapidly develop resistance to these drugs. It has been previously shown that EC19 and EC23, two EC-synthetic retinoids, have single-agent preclinical anticancer activity in colorectal carcinoma. Here, isobologram analysis revealed that they have synergistic cytotoxicity with retinoic acid receptor (RAR) isoform-selective agonistic retinoids such as AC261066 (RARβ2-selective agonist) and CD437 (RARγ-selective agonist) in Caco-2 cells. This synergism was confirmed by calculating the combination index (lower than 1) and the dose reduction index (higher than 1). Flow cytometry of combinatorial IC_50_ (the concentration causing 50% cell death) confirmed the cell cycle arrest at the SubG_0_-G_1_ phase with potentiated apoptotic and necrotic effects. The reported synergistic anticancer activity can be attributed to their ability to reduce the expression of ATP-binding cassette (ABC) transporters including P-glycoprotein (P-gp1), breast cancer resistance protein (BCRP) and multi-drug resistance-associated protein-1 (MRP1) and Heat Shock Protein 70 (Hsp70). This adds up to the apoptosis-promoting activity of EC19 and EC23, as shown by the increased Caspase-3/7 activities and DNA fragmentation leading to DNA double-strand breaks. This study sheds the light on the possible use of EC-synthetic retinoids in the rescue of multi-drug resistance in colorectal cancer using Caco-2 as a model and suggests new promising combinations between different synthetic retinoids. The current in vitro results pave the way for future studies on these compounds as possible cures for colorectal carcinoma.

## 1. Introduction

Chemotherapeutic resistance is one of the main challenges facing drug discovery, where it is responsible for more than 90% of cancer-related deaths [1]. Multi-drug resistance (MDR) emerging from cancer cells occurs through various mechanisms, including the increased efficiency of DNA damage repair [2], the exaggerated intracellular metabolism of drugs [3], genetic and epigenetic alternations [4,5,6] and the enhanced efflux of xenobiotics [7,8]. Therefore, there is an urgent need to find new alternative targets to diminish anti-cancer resistance. A class of these targets is ATP-binding cassette (ABC) transporters which are ATPase family members with various distinct structures and functions, and they are necessary for both normal and cancer cells [9,10,11,12]. ATPase family members comprise several ABC transport systems such as P-type ATPases, V-type ATPases, kinesins, helicases, heat-shock proteins and ATPases associated with different cellular activities (AAA-ATPases) [9]. ATPases were shown to be highly associated with MDR in many types of cancer [12,13,14,15,16]. These ATP-requiring enzymes pump various classes of anti-cancer compounds out of the cell, reducing their intracellular levels, bioavailability and, consequently, their therapeutic potency [17,18]. Therefore, the inhibition of the pumping activity of these enzymes often leads to a higher cellular concentration of the chemotherapeutic drugs, and thus a greater anti-tumor effect [19]. The ABC transporters include P-glycoprotein 1 (P-gp1, MDR1 or ABCB1) [14], multi-drug resistance-associated protein-1 (MRP1 or ABCC1) [15,16] and breast cancer resistance protein (BCRP or ABCG2) [12]. They are associated with MDR in various cancers [20,21,22]. The perturbations in the gene expression of ABC enzymes have been documented in various cancers, leading to drastic changes in the signaling of many ions and molecules promoting tumorigenesis [9]. This reinforces ABC family members, especially P-gp1, BCRP and MRP1, as attractive targets for combating cancer. The addition of efflux-pump inhibitors to anticancer agents exaggerates their activity to improve patients’ responses to chemotherapy [11]. Various small molecule compounds have been tested to modulate or inhibit the activity of P-gp1, BCRP and MRP1 such as Cyclosporine A, Elacridar, Tariquidar, Zosuquidar and Bepridil [23,24]. However, many of these molecules failed in clinical trials.

Retinoids are vitamin A derivatives which regulate the expression of many target genes, including ABC genes, upon interaction with their specific nuclear receptors [25,26,27]. They play a vital role in mammalian physiology during infancy and adulthood [28]. They influence many signaling pathways by spanning several trans-acting, DNA-binding receptors. This family of nuclear receptors has two subfamilies: retinoic acid receptors (RARs) and retinoid X receptors (RXRs). Each receptor subfamily includes three isotypes: RA(X)Rα, RA(X)Rβ and RA(X)Rγ [29]. Moreover, the alternative mRNA splicing and differential promoter activation generate several isoforms from each isotype [30]. The parent natural ligand for RARs is all-*trans*-retinoic acid (ATRA), the main natural intracellular metabolite of vitamin A [31]. Additionally, there are two other natural active analogues originated by intracellular isomerization of ATRA called 9-*cis*-RA and 13-*cis*-retinoic acid (13-*cis*-RA; isotretinoin) that are the prototype ligands for both RARs and RXRs [32]. ATRA acts as a pan-agonist of RARs and activates heterodimerizations with RXRs, PPAR-γ, estrogen receptors or other nuclear receptors, and the active complex binds to the retinoic acid response elements and initiates the transcription of several retinoic acid-targeted genes through a RAR-dependent mechanism [33]. Interestingly, retinoids are important for diverse biological processes such as embryonic morphogenesis, cell growth and hematopoiesis in vitro and in vivo [34]. It has been previously shown that retinol, all-*trans*-retinoic acid (ATRA) 13-cis-RA and retinyl-acetate could interfere with P-gp1 and BCRP activity [35,36]. During pregnancy, some retinoids show uneven distribution between the placenta and the embryo, indicating that placental active transporters, similar to P-gp1, BCRP and MRP1, may be involved in their transport in order to protect the fetus from the toxic accumulation of high levels of retinoids [37,38]. A low intracellular retinoid concentration was observed in P-gp1-induced retinoid-resistant leukemia cells, emphasizing the role of P-gp1 in retinoid resistance [29,32,33]. Retinoid-resistant ovarian cells were rendered sensitive to ATRA treatment after suppressing P-gp1 and BCRP expression [39]. Based on these observations, investigators have suggested that there may be an interaction-mediated crosstalk existing between the drug efflux pumps and retinoids inhibitors of P-gp1, BCRP and MRP1 that may improve the response to retinoids in cancer [40,41,42].

ATRA was used by the Whiting group as a scaffold to synthesize two chemical derivatives called EC-synthetic retinoids have been synthesized [43]. They have been tested for their biological activities on a wide range of cells including stem cells and cancer cells [44,45,46]. The *para*-isomer, 4-(5, 5, 8, 8-tetramethyl-5, 6, 7, 8-tetrahydronaphthalen-2-ylethynyl) benzoic acid, has been given the name EC23 while the *meta*-isomer, 3-(5, 5, 8, 8-tetramethyl-5, 6, 7, 8-tetrahydronaphthalen-2-ylethynyl) benzoic acid, is called EC19. The two analogues showed higher physicochemical stability for extended periods in the laboratory environment [43]. The data showed the differential activities of these molecules on stem cells and cancer cells by the induction of cellular differentiation on a molecular level through the stimulation of RARs [45], leading to evident anticancer activity in sub-molar doses [46]. In addition, these molecules have a synergistic effect with other chemotherapeutic agents such as 5-fluorouracil in Caco-2 cancer cells [44]. In a previous report [44], we performed a general screening of the anticancer activity of EC19 and EC23 on a panel of cell lines, and Caco-2 was among the most sensitive cell lines. Upon reaching cellular confluency, the cells show characteristics of enterocytic differentiation [47]. Notably, Caco-2 cells express retinoic acid-binding protein I and retinol-binding protein II, which represent an integral part of retinoid signaling [48,49]. This makes Caco-2 an ideal model for studying retinoid pathways in colorectal carcinoma. The aim of this study is to further investigate the anti-cancer activity of EC19 and EC23 on RAR-dependent and independent levels through an in vitro approach, including DNA damage activity and interference with the expression/activity of the efflux enzymes and transporters in the Caco-2 cell model. EC19 and EC23 have been combined with other retinoids of known anti-cancer activity targeting either RAR-β or RAR-γ specifically to determine the combinatorial effect to eradicate cancer cells and to “evade” the development of future resistance. Of note, the stimulation of RAR-β and RAR-γ signaling pathways have been linked to the activity of EC-synthetic retinoids and strongly linked to the demise of colorectal cancer cells [44,50]. Overall, this study is expected to enrich our knowledge about the potential use of EC-synthetic retinoids to diminish anti-cancer resistance and to understand their mechanisms of action through new targets in colorectal cancer (CRC).

## 2. Results

### 2.1. Anti-Proliferative Activity of Different Synthetic Retinoids on Caco-2 Cell Line (Individually and in Combinations)

First, the antiproliferative potential of the individual retinoids was screened using the MTT viability assay, and the IC_50_ values after 24 h were calculated (Table 1). EC19 and EC23 were more potent than their parent compound ATRA (IC_50_ values: 27.20 ± 1.8, 23.00 ± 1.2 and 97.70 ± 9.0 µM, respectively) and they showed comparable IC_50_ values as expected from our previous study on Caco-2 cells [44]. The selective RAR-β agonist (AC261066) showed a comparable IC_50_ value (26.90 ± 2.1 µM) to that of EC-synthetic retinoids while the selective RAR-γ (CD437) was the most potent, with an IC_50_ value of 2.80 ± 0.7 µM.

The combinations with RAR-agonists using 10, 5, 2, 1, 0.5, 0.25× (times the IC_50_) dilutions of agonists with ATRA, EC-19 and EC-23 have been carried out and all the combination parameters ×IC_50_, IC_50_ doses after combinations, DRI and CI) have been calculated. The results showed the combination strategies between ATRA and EC-synthetic retinoids with either CD437 or AC261066. The collective IC_50_ of ATRA with CD437 was 5.8 ± 1.2 µM, showing higher IC_50_ values than single CD437 treatments (Table 1). This was confirmed by a lower DRI value than 1 (i.e., 0.48) and a higher CI than 1 (i.e., 4.1), suggesting the combination is not effective. On the other side, the agonistic combinations between both EC-synthetic retinoids and CD437 were more potent and significant where the resulting collective IC_50_ values were lower than 1 µM, with a preferential effect shown by EC19 (<0.1 µM). This was reflected in higher DRI values (24.95 for EC19 and 3.45 for EC23) and lower CI than 1 (0.1 for EC19 and 0.6 for EC23), suggesting that the combination strategy is more effective (especially with EC19). The same scenario has been observed for the combination with AC261066, which showed a preferential combination in the case of EC19 followed by EC23 and then ATRA (Table 1).

Finally, the combinations between ATRA or EC-synthetic retinoids with the selective RAR-β/γ antagonist CD2665 were tested regarding the activity and potency of EC-compounds through RARs. First, Caco-2 cells were treated with CD2665 in combination with ATRA or EC-synthetic retinoids for 24 h, and the data showed that ATRA was preferentially able to ameliorate the effect of RARs blockage mediated by CD2665 with a considerably lower combined IC_50_ value (5.86 µM), higher DRI value (16.67) and lower CI value (0.12) (Table 1). Second, the effect of CD2665 was tested for 8 h, and then the culture media was washed out, and finally, ATRA or EC-synthetic retinoids were added for an additional 16 h (total exposure for 24 h). The data showed that ATRA was not able to ameliorate the effect of CD2665 where a higher IC_50_ value after combination was observed compared to about double the value (215.04 µM) along with a non-effective combination (low DRI (0.45) and high CI (8.3)). In contrast, EC19 and EC23 were able to ameliorate the blockage effect of RARs with a 10-fold reduction in their IC_50_ values after combination (EC19: 2.88 µM and EC23: 3.52 µM) and the favorable parameters of the combination (DRI value (9.44 for EC19 and 6.53 for EC23) as well as a lower CI than 1 (0.2 for EC19 and 0.3 for EC23)) showing a preferential effect to EC19 (Table 1).

### 2.2. Apoptotic and Cell Cycle Analysis of Retinoids in Individual and Combinatorial Effect

Since EC-synthetic compounds previously showed significant apoptotic effects and interference with the cell cycle progression on Caco-2 cells, it was interesting to examine the combinatorial effects of ATRA or EC-synthetic compounds with either CD437 or AC261066. In Table 2, the data showed that individual IC_50_ doses previously calculated of ATRA or EC-synthetic retinoids were able to significantly decrease the percentage of viable cells with a preferential effect to EC19 (64.80% ± 3.5) compared to control (98.11% ± 5.6; *p =* 0.0214). Moreover, all retinoids significantly increased the percentages of both early apoptotic and necrotic cells (*p* < 0.001), and EC19 was additionally capable of increasing the percentage of late apoptosis (5.06 ± 1.2*) (*p* = 0.025). Additionally, individual IC_50_ doses of CD437 and AC261066 showed similar patterns to EC-synthetic retinoids and were able to significantly increase the early apoptotic cell percentage (17.42% and 27.40%), respectively, and increase the percentage of late necrotic cell percentage (12.84% and 4.12%), respectively. After combination with either CD437 or AC261066 using the combined IC_50_ doses of each retinoid as previously calculated, all retinoids showed a significant reduction in the percentage of viable cells (*p =* 0.0075) and increment in all percentages of early, apoptotic and necrotic cells (*p <* 0.001) reinforcing the preferential effect to the combinations containing EC19 (Table 2).

Regarding the cell cycle analysis, Table 3 shows that the control viable cells had a homogenous distribution of cells between the G_0_-G_1_ phase (53.46% ± 3.9), S-phase (13.01% ± 1.3) and G_2_M phase (33.07% ± 2.2). After 24 h treatment with individual retinoids, ATRA was able to significantly reduce the percentage of dividing cells in the G_0_-G_1_ phase (0.67%) (*p <* 0.001) and induce cell arrest at the G_2_M phase (94.73% ± 5.4) (*p <* 0.001). Additionally, EC-synthetic retinoids were able to arrest most of the cells at the G_2_M phase with a reduction in dividing cells at the G_0_-G_1_ phase (Table 3). The agonist CD437 and AC261066 were able to significantly reduce the percentage of cells at the G0-G1 phase (3.78% and 2.20%), respectively, and increase the percentage of cells arrested at the G_2_M phase (86.99% and 90.16%), respectively, in a way similar to EC-synthetic retinoids. In addition, all combinations showed significant cell cycle arrest at the sub-G_0_-G_1_ phase (*p* < 0.001); the phase where cells are preparing to enter cell cycle propagation with a reduction in the G_2_M phase. EC19 had the most preferential effect either individually or in combinations (Table 3).

### 2.3. Retinoids Induce Caspase-3/7 Activity with Detectable Apoptosis and Necrosis in Caco-2

**Figure 1:**
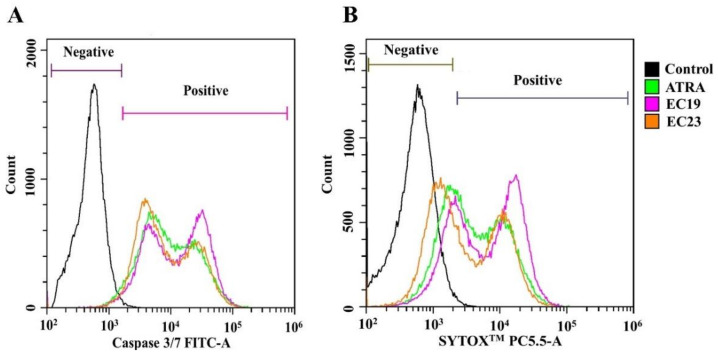
Overlay histogram of Caco-2 cell population detected with Caspase-3/7 Green and SYTOX™ Flow Cytometry Assay Kit after exposure to retinoids. The cells were manipulated with the IC_50_ dose of ATRA or EC-synthetic retinoids for 24 h. Negative control is cells treated with 0.1%DMSO. Both (**A**) the CellEvent™ Caspase-3/7 Green Detection Reagent and (**B**) the SYTOX™ AADvanced™ Dead Cell Stain were used to detect apoptotic and necrotic populations, respectively. The samples were analyzed by Cytoflex flow cytometer using 530/30 and 690/50 bandpass filters, respectively. Retinoids induced more staining with both dyes causing population shift in comparison to the untreated control, reflecting the activation of both apoptosis and necrosis. Shown are representative histograms of three independent experiments (*n* = 3).

Necrosis has been noticed from the apoptosis assay, and it was essential to confirm the apoptosis/necrosis effect using specific markers. Herein, we measured the activity of both Caspase-3/7 as well as the necrotic activity inside the cells after 24 h of treatment using flow cytometry. SYTOX™ is a new and powerful high-affinity nucleic acid stain that can be used to discriminate dead necrotic cells using the common 488 nm blue laser in flow cytometry. Table 4 shows the distribution of live, necrotic and apoptotic populations of Caco-2 cells treated with individual retinoids compared to solvent control. All retinoids caused a significant reduction in live cells compared to 0.1% DMSO-treated control (** *p* < = 0.009). ATRA caused the cell population to shift towards apoptosis (11.75 % ± 2.92) and necrosis (82.69% ± 7.13) events in comparison to 1.0.1% DMSO control (74% ± 1.22 and 10.75% ± 1.69) (* *p* = 0.042). When the cells were exposed to EC19 and EC23, a similar pattern was observed, and most cells became either apoptotic or necrotic, as revealed by the overlay histogram (Figure 1). The significant increment in Caspase-3/7 and SYTOX™ activities and the activation of the necrosis pathway were confirmed by the calculation of apoptotic and necrosis percentages, respectively. Table 4 reveals that higher apoptotic and necrotic cells were detected after 24 h treatment with all retinoids. The apoptotic and necrotic cell percentages were obtained by calculating the percentage ratio of apoptotic and necrotic cell events, respectively, to the whole cell population according to the standard protocol [51,52]. These results come in line with the observed pro-apoptotic activity of EC-synthetic retinoids shown in our previous study and apoptotic assay [44] and reinforce the ability of EC-synthetic retinoids to boost the Caspase cascade on transcriptomic, proteomic and post-translational levels. Furthermore, we showed here that these compounds can also activate cell necrosis leading to the observed reduction in cellular viability.

### 2.4. EC19 and EC23 Interfere with the ATP-Catabolizing Activity of Calcium-Independent ATPases in Caco-2 Cells

**Figure 2:**
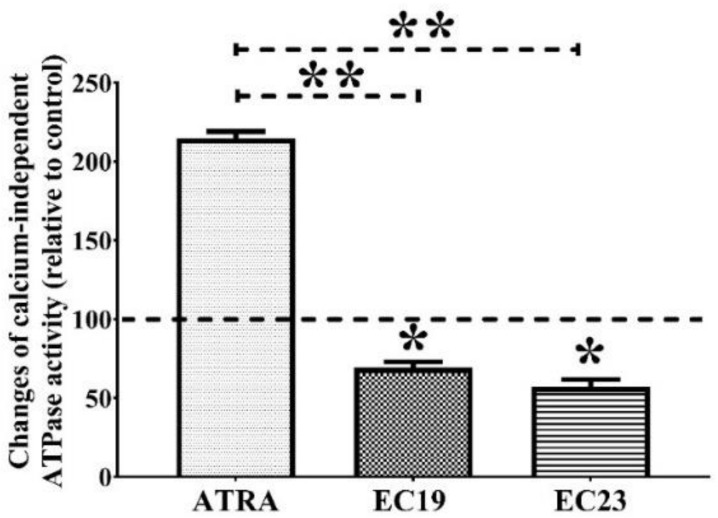
The total activity of calcium-independent ATPases after the exposure of Caco-2 cells to retinoids. Caco-2 cells were treated with the IC_50_ concentration of the indicated retinoids for 24 h and the enzymatic activity was measured colorimetrically. The ATPase activity observed in 0.1% DMSO control was normalized to 100% (the dotted line represents the control). The activity in presence of EC-synthetic retinoids was then detected and compared to the control and ATRA. Shown is the mean change in ATP-degrading activity ± SEM of three independent experiments (*n* = 3). * *p* < 0.05 (compared to the control) and ** *p* < 0.01 (compared to ATRA) indicated statistically significant difference.

The effect of individual IC_50_ doses of EC-synthetic retinoids on the activity of total calcium-independent ATPases in Caco-2 whole lysate was investigated, and the amount of inorganic phosphate (Pi) liberated from ATPase enzymes was calculated and compared to the solvent control and the individual IC_50_ dose of the parent ATRA after 24 h. While ATPase activity was surprisingly increased with ATRA, the data showed that EC19 and EC23 were able to reduce the liberated Pi by approximately 30% and 43%, (* *p* = 0.041), (* *p* = 0.039), respectively, in comparison to the negative control (Figure 2). The ATPase activity in the presence of EC-synthetic retinoids was markedly lower than the enzymatic activity after ATRA treatment (** *p* = 0.006). Although the whole cellular lysate comprises a wide variety of distinct ATPases, the ability of retinoids to suppress the enzymatic ATP-harnessing capacity suggests a possible multi-target action that needs to be further investigated.

### 2.5. EC19 and EC23 Induce DNA Fragmentation and Genotoxicity in Caco-2 Cells

**Figure 3:**
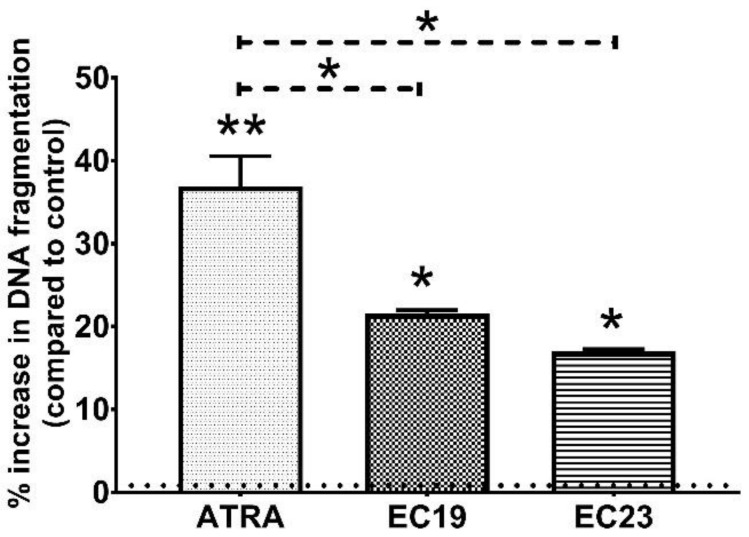
Retinoid-induced DNA fragmentation in Caco-2 cells after 24 h. The cells were treated with the IC_50_ concentrations of ATRA, EC19 or EC23. The DNA fragmentation after treatment was quantified colorimetrically as the relative percentage of fragmented DNA to the total DNA content in each sample compared to 0.1% DMSO control (dotted line) and depicted as the mean percent increase in DNA fragmentation ± SEM of three independent experiments (*n* = 3). All retinoids induced significant DNA fragmentation in comparison to ATRA highlighting their genotoxicity in Caco-2 cells. * *p* < 0.05 and ** *p* < 0.01 indicate statistically significant difference.

DNA fragmentation is a hallmark of apoptosis detectable after Caspase cascade activation, it was measured using the colorimetric diphenylamine assay after the exposure to individual retinoids [53,54,55]. Figure 3 showed that the individual IC_50_ dose of ATRA caused a 36.83% ± 3.72 increase in DNA fragmentation in comparison to the 0.1% DMSO control (** *p* = 0.005). Interestingly, individual IC_50_ doses of EC19 and EC23 also induced DNA fragmentation with 21.53% ± 0.45 and 16.95% ± 0.31 more than the control (* *p* = 0.039), respectively. These results reinforce the characteristic hallmarks of apoptosis that have been observed.

### 2.6. Confirmation of DNA Damage Effect of EC-Synthetic Retinoids Using Advanced Immunocytochemistry Analysis of Phosphorylated H2AX (HCS DNA Damage)

**Figure 4:**
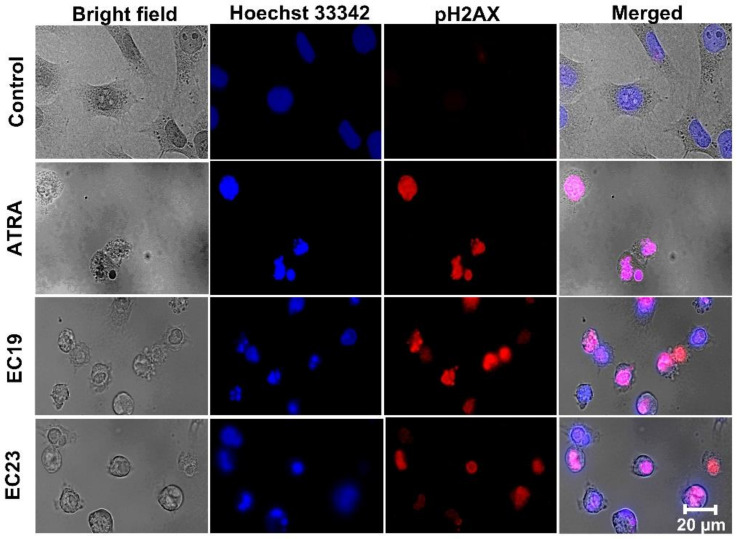
Imaging and analysis of genotoxicity induced by EC-synthetic retinoids in Caco-2 cells using the HCS DNA Damage Kit. Caco-2 cells were treated with IC_50_ concentration of EC-synthetic retinoids as well as ATRA compared to the negative control (0.1%DMSO) for 24 h at 37 °C, 5% CO_2_. Using the Carl Zeiss LSM 710 confocal microscope (63× magnification; at 20 µm scale), these images were captured to clearly indicate that many γ-H2AX foci were observed in the Caco-2 cells treated with all retinoids with preference to EC19, but not with negative control. Hoechst 33,342 was used to map nuclei.

Given the observed cell viability results and the anti-cancer activity of EC-synthetic retinoids, we investigated the genetic toxic effect of EC19 and EC23 in the treated Caco-2 cells in comparison to ATRA. We used the HCS DNA Damage Kit (Invitrogen) which enables the simultaneous quantitation of two cell health parameters: genotoxicity and cytotoxicity. The phosphorylated H2AX (γH2AX) foci formed at the damage site in the nucleus are a biomarker of DNA-single breaks (DSBs), which were measured by specific antibody-based detection. Toxicity was also measured with the Image-iT^®^ DEAD Green™ viability stain. The assay kit also included Hoechst 33342 (a DNA-binding dye emitting blue fluorescence), which showed the nuclear morphology of all intact and damaged cells. Figure 4 shows the fluorescence images of the treated Caco-2 cells and the DSBs after treatment with the IC_50_ dose of EC- retinoids concentration. There was only an increase in DSB yield in both ATRA and EC-synthetic retinoids with a preference for EC19. Moreover, the cell images showed serious cell injuries in the treated Caco-2 cells. These results are consistent with the cell viability results and DNA fragmentation assay shown in Figure 3. Therefore it was concluded that EC-synthetic retinoids are highly toxic and effective in inducing DNA DSBs in Caco-2 cells.

### 2.7. EC19 and EC23 Modulate the Chemoresistance-Related Genes and Proteins and Caco-2-Supporting ATPases Levels

**Figure 5:**
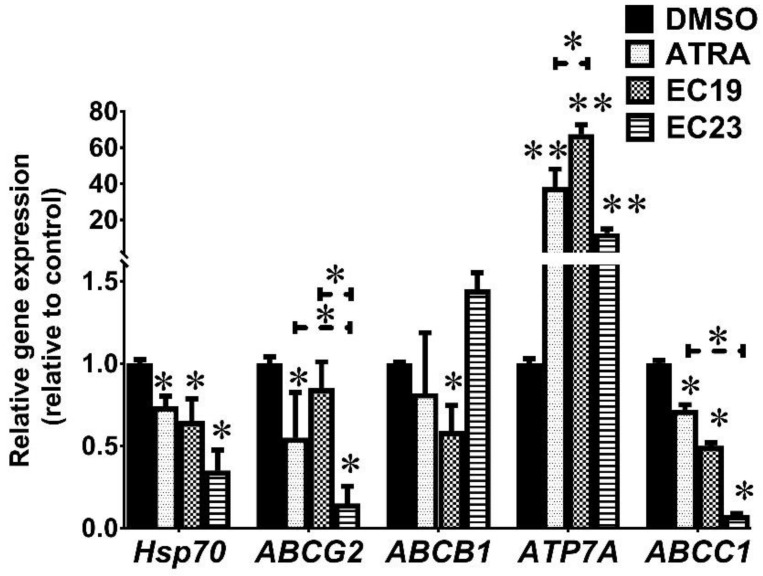
Gene expression analysis using RT-qPCR of retinoid-treated Caco-2 cells. The cells were treated with the IC_50_ concentrations of retinoids for 24 h and the qPCR reactions were carried out using HERA^PLUS^ SYBR^®^ Green qPCR Kit. The differential gene expression of the indicated genes was performed using the 2^−ΔΔCT^ method in comparison to the negative control (0.1%DMSO). Shown is the mean ± SEM of three independent experiments (*n* = 3). * *p* < 0.05 and ** *p* < 0.01 indicate statistically significant difference compared to negative control.

It was essential to identify which ATPases have been depleted after retinoid treatment for 24 h. We used RT-qPCR to detect mRNA expression of *ABCB1* (which codes for P-gp1 protein), *ABCG2* (which codes for BCRP protein), *ABCC1* (which codes for MRP1 protein), *ATP7A* (which is a copper efflux ATPase pump) and Heat Shock Protein 70 (*Hsp70*). The results showed that the expression of both *Hsp70* and *ABCC1* has been significantly reduced by the tested retinoids (* *p* = 0.044) (Figure 5). At the same time, all retinoids caused a profound upsurge in the level of *ATP7A* with more than 38-folds in comparison to the 0.1% DMSO control (** *p* = 0.006). Each retinoid had a characteristic effect on the expression of the rest of the detected ABC genes. In the case of ATRA, the expression of *ABCG2* was downregulated to 0.55 ± 0.28-folds compared to the control (* *p* = 0.037), while the expression of *ABCB1* was not significantly different (*p* = 0.09). In the case of EC19, *ABCB1* expression was reduced to the least value after 24 h (0.59 ± 0.16 folds; * *p* = 0.038) whereas *ABCG2* expression was not significantly downregulated compared to the control (*p* < 0.05). Interestingly, EC23-treated cells showed lower *ABCG2* expression than ATRA and EC19 (* *p* = 0.04), reaching a minimal level of 0.15 ± 0.11 folds in comparison to the solvent control (* *p* = 0.036). In summary, our results showed that EC-synthetic retinoids can reduce the expression of multiple ABC transporter genes as well as *Hsp70*.

**Figure 6:**
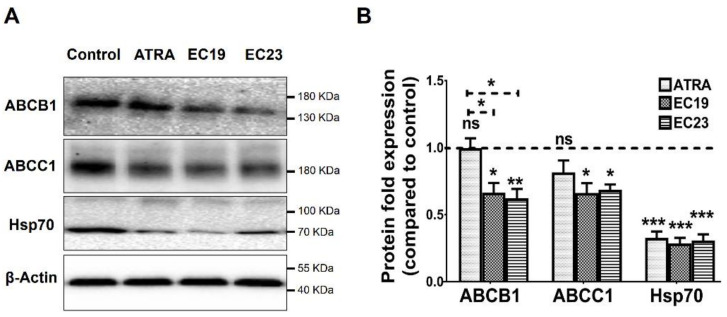
Western blotting analysis of ABCB1 (*MDR1*), ABCC1 (*MRP1*) and Hsp70 in Caco-2 cells. (**A**) Representative immunoblotting images demonstrate the effect of IC_50_ dose of ATRA, EC19, and EC23 on the expression levels of the indicated proteins in Caco-2 cells treated for 24 h. The expression of the investigated proteins in treated Caco-2 cells was calculated and normalized to the β-actin protein level in each treated sample, and finally plotted as (**B**) the mean protein fold change from negative control (0.1%DMSO) (dotted line) ± SEM. It can be clearly seen that EC19 and E23 caused significant downregulation of all proteins in comparison to the 0.1% DMSO control. * *p* < 0.05, ** *p* < 0.01 and *** *p* < 0.001 indicate a statistically significant difference. ns; non-significant.

On the protein level, Western blotting showed interesting results (Figure 6). All retinoids were able to significantly reduce Hsp70 protein levels (*** *p* < 0.001). For MDR1 (i.e., *ABCB1*) and MRP (*ABCC1*) proteins, ATRA did not induce any significant change in their levels, unlike the EC-retinoids. While EC19 showed significant reduction in their levels by about 34% compared to the solvent control (* *p* = 0.039), EC23 caused significant downregulation of MDR1 and MRP by 38% (** *p* = 0.008) and 32% (* *p* = 0.021), respectively. Interestingly, the EC-retinoid-induced downregulation of MDR1 protein was more significant than the parent ATRA (* *p* = 0.033). Collectively, EC19 and EC23 suppress ABC and Hsp70 expression on mRNA and protein levels.

## 3. Discussion

### 3.1. The Potential Application for Anti-Cancer Activity of EC-Synthetic Retinoids

Despite the efficiency of many anti-cancer drugs, including retinoids such as ATRA, in suppressing Caco-2 cell growth [44,45,46], the chronic administration of these compounds for colorectal carcinoma patients may lead to serious adverse effects and the development of anti-cancer resistance [56]. Therefore, there is an urgent need to use new synthetic compounds with subsequent low active doses and diverse molecular targets. EC-synthetic retinoids were shown to have potent in vitro anti-cancer activity on a wide range of cancer cell lines including Caco-2 cells with minimal cytotoxicity to normal cells [44]. These synthetic retinoids were shown to have a pro-apoptotic effect by activating caspases and cytochrome-C with cell cycle arrest [44]. The anti-cancer effect of these compounds was attracting to test their activity on other non-RAR molecular targets with the possibility of incorporating them in combination with other anti-cancer retinoids. This will achieve three main goals: first, synergistic cytotoxicity that allows the usage of markedly lower doses of both agents. Secondly, reducing the side effect resulting from high doses of non-selective RAR agonists and finally, evading the emergence of resistance of cancer cells to the other retinoids to improve the pharmacokinetics and increase the intracellular levels of the combined retinoids.

### 3.2. RAR-Dependent Activity of EC-Synthetic Retinoids

It was shown that both EC19 and EC23 have synergistic cytotoxicity with RAR isoform-selective agonistic retinoids (CD437 and AC261066) in Caco-2 cells. AC261066 is an orally active selective RAR-β_2_ agonist, which linked the RAR-β_2_ stimulation with isoform-dependent mammalian tumor suppression [57]. CD437 is a RAR-γ agonist with promising activity for the treatment of various types of cancers including Caco-2 cells [58]. Herein, we found that the combined IC_50_ of AC261066 or CD437 with EC19 or EC23 was significantly lower than their individual IC_50_ concentrations with a preferential effect to EC19. This can be explained based on a receptor binding assay study on the level of the RAR molecular target as both EC19 and EC23 have higher binding affinity to RAR-β, and EC23 has high RAR-α affinity than the parent ATRA [46]. Therefore, the combined RAR-β and RAR-α have been shown to have the synergistic and potent inhibition of Caco-2 cells [50,59,60].

Challenging Caco-2 cells with the Selective RARβ/γ antagonist CD2665 was important here to see whether the activity of EC-synthetic retinoids is RAR-mediated activity, and whether the inhibition of RARs for short and long terms would rescue the retinoid-treated Caco-2 cells or not. Although CD2665 has been long described as an antagonist for RAR-β and RAR-γ with binding constant K_d_ values of 306 and 110 nM, respectively [61], recent reports showed that CD2665 has a high affinity to the three RAR isotypes at the micromolar level [62]. In the present study, CD2665 was able to suppress the growth-inhibitory activity of EC-synthetic retinoids at the long-term combinations for 24 h, with combined IC_50_ values similar to the individual IC_50_ values of EC-synthetic retinoids. On the other side, the short-term RARs blockage for 8 h with CD2665 was abolished after washing out, and continuous treatment with EC-synthetic retinoids resulted in combined IC_50_ values lower than those of individual or long-term combinations. This observation suggests the possible involvement of RAR-dependent cellular pathways in mediating EC-synthetic retinoid actions [63]. The data outcome fits into the context of the present literature [62,64].

### 3.3. EC-Synthetic Retinoids Induce Cellular Apoptosis, Necrosis, and Cell Cycle Arrest Individually and in Synergistic Combinations

The previously observed synergistic activity of EC-synthetic retinoids with RAR-agonists was confirmed by the flow cytometric analysis through the induction of cellular apoptosis. CD437 and AC261066 were shown to induce cellular apoptosis in some cancer cell lines such as colorectal [65], esophageal squamous [66] and Thymocytes [61] through RAR-dependent mechanisms. The combination of EC-synthetic retinoids with these agonists had a potent effect that was observed with additional necrotic cells that are positive for Annexin/PI dyes. This observation has been documented in the literature for synthetic retinoid combinations [67,68,69] and explained by the induction of DNA-damaging that blocks DNA replication, causing the collapse of replication forks and double-strand breaks (DSBs) [70,71]. DSBs induce the p53-independent activation of necrosis which is mediated maybe by the death domain kinase, RIPK1 (receptor-interacting protein kinase-1) [72,73,74] that needs to be further investigated. Necroptosis is a type of programmed, caspase-independent cell death that is morphologically similar to the necrosis activated by the tumor necrosis factor (TNF), the receptor interacting with the protein kinases, RIPK1 and RIPK3, and the mixed lineage kinase domain-like (MLKL) [75,76]. The DNA-damaging effect induces non-apoptotic death such as necroptosis and pyroptosis; however, the detailed mechanism is still elusive [77].

On the level of cell cycle analysis, the individual retinoids induced significant cell cycle arrest at the G_2_M phase, only affecting the mitogen-activated protein kinase and cyclin-dependent kinase, which are commonly observed with natural and synthetic retinoids [69,78,79,80,81]. The combinatorial effect of EC-synthetic retinoids with RAR-specific agonists induced additional significant accumulation of cells at the sub-G0-G1 phase, with a reduction in G_2_M phase indicating that the cellular DNA had begun to fragment as a biomarker of necrotic cell death [82]. The previous reports suggest that retinoids can induce cell cycle arrest at different phases depending on the concentration of the retinoids [83], the degree of DNA fragmentation [83,84], the retinoids’ combination effect [85,86] and the activity of the retinoids on different cell cycle checkpoint-controlling proteins [87]. 

Interpreting the results from the standard apoptosis assay and the recent CellEvent™ Caspase-3/7 Green, SYTOX™ is very useful for the detection of the later stages of cell death. This was confirmed by the binding of the annexin-V to “flipped” PS residues indicating the early stage of apoptosis, and cell membrane integrity using DNA binding dyes indicating the late stage of apoptosis [88]. Apoptosis is a silent form of cell death while necrosis causes the stimulation of the immune system leading to the acute inflammatory state mediated by some mediators such as TLR [89] and Gasdermin [90,91].

### 3.4. EC-Synthetic Retinoids like Other Retinoids May Mediate Their Activities through Additional RAR-Independent Pathways

To understand the molecular mechanism of the effects of EC-synthetic retinoids and their potency compared to ATRA on the genetic level of Caco-2 cells, it was suggested possible involvement of other RAR-independent pathways in mediating their actions. This fits into the context using some examples from synthetic retinoids in the literature [63,64]. Although the cellular response to CD437 was previously thought to be only RAR-γ-dependent [63], the anti-tumor effects in the Caco-2 cells appeared to be mediated through other various RAR-independent targets [64]. Several lines of evidence further support this observation. First, the co-incubation of CD437 with the RAR pan-antagonist did not inhibit cytotoxicity in Caco-2 cells with various agonists [92,93]. Second, mutations in the gene encoding DNA polymerase α (known as *POLA1*) led to the resistance of colorectal cancer to CD437 and its analogues, regardless of the RAR signaling status [65]. Third, SAR studies involving CD437 did not find a significant correlation between the ability of the compound to activate RAR-γ and cytotoxicity [94]. Finally, some types of leukemia that lack RAR expression remained sensitive to CD437 [79].

Another example is the inability of CD2665 to inhibit retinoid-induced Caco-2 cell death can be through the RAR–RXR heterodimer formation. In a previous study, the synergistic growth inhibitory effect has been observed by the combination of CD2665 with RXR agonists (e.g., CD3254) through RAR–RXR heterodimer formation [62]. RAR-α binding by CD2665 was revealed to play a role in this effect through RAR-RXR heterodimer formation. When CD2665 binds to a RAR-α ligand-binding domain (LBD), it leads to the subsequent dissociation of the co-repressor SMRT. Then, the binding of a retinoid with a high affinity to RXR allows the recruitment of the co-activator TIF2 initiating the RAR-α-RXR-mediated genetic program. The activation of this program may have led to a reduction in the cellular growth of the Caco-2 cancer cell line in a manner that is similar to the individual treatment with RAR agonists. The similarity in growth inhibition in the consequence of multiple RAR activation or RAR-α-RXR heterodimerization may be attributable to SMRT dissociation and replacement with TIF2 [62,95]. It seems that the results from binding affinity studies using only RAR-dependent activity cannot be generalized on cell-based assays, and the levels of other pathways should be investigated to further improve our understanding of EC-synthetic retinoid mechanisms.

#### 3.4.1. EC-Synthetic Retinoids Reduce ATPase Activity

ATP7A expression is one of the hallmarks in the progression of Caco-2 cells, carcinogenesis and resistance to chemotherapy [96,97,98,99]. ATP7A is a copper efflux ATPase pump that pumps copper outside Caco-2 cells. This may add up to the anticancer potency of EC19 and EC23 possibly by exhausting the cellular stores of copper and interrupting the copper-dependent signaling pathways. While some reports correlated ATP7A expression with growth inhibition and cell death in Caco-2 cells, others suggested that ATP7A upregulation is associated with tumorigenesis, malignancy and MDR [19,100,101,102]. The gene coding for ATP7A is a retinoid-responsive gene whose expression is tissue-dependent in both normal and malignant states. A cross-talk was previously documented between the ATP7A and RAR-β isoforms [103]. The ATP7A expression was induced after the ectopic overexpression of RAR-β_2_, and thus the silencing of RAR-β_2_ with siRNA blocked the upregulation of ATP7A in retinoid-treated cells [103]. The forced downregulation of RAR-β_2_, and subsequently ATP7A, reduced copper efflux and increased the viability of retinoid-treated Caco-2 cells. This hypothesis matched with the observed activity of EC-synthetic retinoids to induce RAR-β activity on both receptor binding and cellular assays [44,46].

#### 3.4.2. EC-Synthetic Retinoids Induce DNA Fragmentation in Caco-2 Cells

Usually, ATRA and other vitamin A supplements do not induce structural modifications in chromosomes of human embryonic mesenchymal cells during differentiation stages [104]. However, some synthetic retinoids have been shown to induce their anti-cancer activity through the induction of DNA double breaks (DDBs), genotoxicity and chromosomal aberrations that remain unrepaired in the next cell generations inducing cellular apoptosis and necrosis [105]. This observation was matched with our data obtained using the sensitive method for the determination of DNA damage by diphenylamine assay, which is characterized as a simple method for the manipulation of any type of cell lines where the resulting colorimetric data is easily quantitated and highly reproducible [106]. The total DNA fragmentation showed that ATRA had the highest degree of genotoxicity followed by EC19 and EC23. However, it was essential to precisely understand the molecular mechanisms causing the DNA fragmentation with the γH2A.X staining assay since the inhibition of the DNA damage response pathway (DDR) was responsible for the induction of carcinogenesis and anti-cancer resistance [107]. We have observed that DNA damage occurred significantly with EC-synthetic compounds within 24 h secondary to apoptosis leading to the stimulation of DNA damage repair (DDR) and consequently the S phase cell arrest, with the phosphorylation of its substrates, H2AX to γH2AX. This observation is matched with our previous report about the anti-cancer activity of EC-synthetic retinoids in Caco-2 cells using cell cycle analysis and their ability to reduce the total anti-oxidant capacity leading to more intracellular reactive oxygen species [44]. The DNA damage effect of EC-synthetic compounds can also account for the observed cell cycle arrest in the sub-G_0_-G_1_ phase with a higher percentage of necrotic cells.

#### 3.4.3. EC-Synthetic Retinoids Modulate the Chemoresistance-Related Genes and Proteins

In the current investigation, we showed that EC-synthetic retinoids reduce the gene expression of the efflux pump transporters: P-gp1, BCRP and MRP1 in Caco-2 cells. These enzymes have been long recognized as members of the ABC transporter family with a broad spectrum of substrate specificity including retinoids [10,108]. A higher expression of P-gp1, BCRP and MRP1 has been demonstrated in chemotherapy-resistant cancer cells, including Caco-2 [12,14,96]. Herein, we demonstrated that EC19 and EC23 suppress the ATP-hydrolyzing activity of calcium-independent ATPases which include the ABC family in Caco-2 cells, suggesting a possible interference with the nuclear binding domains (NBDs) of the enzymes. EC23 induced 1.5-fold gene expression, while on protein expression level, it was shown as downregulated. There are some possible explanations here for such inconsistency. Firstly, the 1.5-fold gene expression is on the borderline for gene over-expression, and it may not be sufficient for the effective induction of the observable protein expression pattern. Second, the mRNA posttranscriptional modifications might have led to mRNA instability and reduced protein translation; third, the protein might have been exposed to ubiquitination and proteasomal cleavage. Finally, the expressed proteins may be subjected to damage and/or degradation by ROS. The significant sequence similarity between P-gp1, BCRP and MRP1 apparently has overlapping drug substrate spectra, explaining why their overexpression is correlated with drug resistance in Caco-2 cells [109,110].

The alternations in P-gp1 activity can affect the efficacy and/or safety of chemotherapeutics with narrow therapeutic indices [111]. In addition, drugs that interfere with P-gp1 expression and/or activity may influence the intracellular levels of other concomitant drugs leading to significant drug–drug interactions [111,112]. This led the drug regulatory authorities, including the US Food and Drug Administration (FDA) and the European Medicines Agency (EMA), to require the determination of the P-gp1 inhibitory potential of new drugs prior to their approval [113,114]. This may explain the previously observed synergistic effect of combining the RAR-agonist with ABC-inhibitory anti-cancer retinoids, such as EC19 and EC23, that can reduce their efflux, resulting in maximal synergistic interference with Caco-2 cell growth. Although it has been suggested in the literature that the inhibition of one abundant ABC transporter (e.g., ABCB1) is sufficient to reduce the growth of colorectal cancer cells [115,116], this was measured in this study. However, it is essential here to find other alternative approaches to confirm the activity of these retinoids and other synthetics required to specifically inhibit ABC transporters to confirm the hypothesis. The concentrations of either natural retinoids or synthetic retinoids used in vitro (approximately 1–20 nM) would inhibit the investigated ABC transporters expressed at various cancer cell lines [117]. In comparison, therapeutic doses of retinoids used for the treatment of cancer in vivo would need sufficiently high local retinoid micro-or milli-molar concentrations in the blood [118,119,120,121]. The inhibition of the ABC transporters may also affect the pharmacokinetics of other co-administered chemotherapeutic drugs by increasing intracellular concentrations, raising the possibility of successful combination regimens. Nevertheless, further animal studies are needed to evaluate the effectiveness and pharmacokinetics of combinations containing EC-synthetic retinoids and other cytotoxic retinoids for the successful healing of colorectal cancer.

In addition to ABC transporters, the abundance of certain ATPase enzymes, including Hsp70 that has been previously associated with resistance to effective chemotherapeutics in Caco-2 cells [122]. The signaling of the heat-shock protein (HSP) family is often linked with Caco-2 cellular apoptosis [123,124,125,126]. Biochemical and structural evidence showed the significance of ATP hydrolysis in the function of HSPs [127,128]. During tumorigenesis, the activity of many HSPs is modified; especially Hsp70, which is the most ubiquitous stress-inducible chaperone [129]. This molecule is upregulated in Caco-2 cells to facilitate the refolding or degradation of proteins necessary for survival and metastasis that are denatured owing to stress [130]. The overexpression of Hsp70 inhibits apoptosis and prevents Caspase cascade activation in colorectal cancer [123,124,125,126]. Due to the strong cytoprotective activity of Hsp70, its cellular content has been inversely correlated with the response to the therapy, and thus high Hsp70 is associated with poor cancer prognosis [112,117,118]. In this study, we found that Hsp70 on both gene and protein levels were significantly reduced after the treatment of Caco-2 cells with EC19 and EC23 for 24 h. This could, at least in part, relieve the restrictions on Caspases’ activity and enhance cellular apoptosis. During apoptosis, pro-apoptotic Bcl-2 family members enhance the mitochondrial outer membrane permeability leading to the release of cytochrome C (Cyt C) into the cytosol [131,132]. Cytosolic Cyt C binds Apaf-1, forming a complex that oligomerizes into heptameric Caspase-9-activating Apoptosome. Mature Apoptosomes either cleave, and thus activate, Caspase-3 and Caspase-7 or form bigger inactive aggregates, depending on the availability of nucleotide dATP/ATP. When dATP is available in the cytoplasm, active executioner Caspase-3 induces Cyt C/dATP-inducible cleavage of both the DNA fragmentation factor 45 (DFF45)/inhibitor of Caspase-activated DNase (ICAD) complex and PARP-1 protein, promoting DNA fragmentation and laddering [131,132]. The Hsp70 chaperone directly binds to Apaf-1 and prevents the recruitment of Caspases to the Apoptosome complex. Thus, Hsp70 suppresses apoptosis by directly associating with Apaf-1 and blocking the assembly of a functional Apoptosome.

The downregulation of Hsp70 in Caco-2 cells after the exposure to the anti-proliferative EC-synthetic retinoids may lead to the enhancement of apoptosome formation with subsequent Caspase-3/7 activation and DNA fragmentation which are key determinants for the ongoing apoptosis observed [133,134]. Despite many characteristics of apoptotic cells that were analyzed by the presented methods, chromatin condensation and nuclear fragmentation remain main hallmarks of apoptotic cells that need to be further studied [135,136,137,138]. We confirmed apoptosis induction after the manipulation of Caco-2 cells with EC-synthetic retinoids alone by detecting the DNA fragmentation along with Caspase-3 and Caspase-7 activation. Synthetic retinoids are known to be a potent inducer of reactive oxygen species (ROS) production, confirmed by the phosphorylation of H2AX [139,140,141], alkali-labile sites formation in DNA [140], DNA single-strand breaks and cell death provoked by oxidative stress. Hsp70 acts as a sensor of changes in the NEDD8 cycle controlled by NEDP1 in response to DNA damage to help cells choose between survival and apoptosis [142]. Therefore, the observed DNA fragmentation after treatment is more likely to be caused by Hsp70 downregulation, apoptosis, necrosis and DNA damage induction and the activation of the Caspase cascade.

## 4. Materials and Methods

### 4.1. Retinoids and Chemical Reagents

The natural retinoid ATRA, in addition to the synthetic retinoids EC19, EC23, CD437 (RAR-γ selective agonist), AC261066 (RARβ-2 agonist) and CD2665 (Selective RAR-β/γ antagonist), were purchased from Tocris Biosciences (UK; purity ≥ 98% (high-performance liquid chromatography, HPLC)). The stock solutions of retinoids were prepared in DMSO (Sigma-Aldrich, St. Louis, MO, USA) to a final concentration of 1 mM and stored at −20 °C. The ATRA stock solution and the aliquots of working concentrations were preserved away from laboratory lights during storage and during the experiments. Malachite green, ammonium molybdate, polyvinyl alcohol and ATP were supplied by Piochem (Giza, Egypt) and stored as guided by the manufacturer.

### 4.2. Cell Culture

Caco-2 cell line was purchased from the cell culture bank in the tissue culture unit at the Holding company for the production of vaccines, sera and drugs (VACSERA, Giza, Egypt) and was maintained in the Center of Scientific Excellence “Helwan Structural Biology Research, (HSBR)”, according to the previous protocol [44].

### 4.3. Antiproliferative Activities of Single Synthetic Retinoids and in Combination

First, the ATRA, EC19, EC23 and RAR isoform-selective agonists, CD437 and AC261066, were added individually, or in combinations, to Caco-2 cells to detect the dose required to kill 50% of the cells (IC_50_) alone or in mixtures after 24 h. Previous and recent reports illustrated that CD2665 acts as a pan-antagonist of the three RAR isotypes at 1 µM concentration within a few hours [62]. We previously showed the antiproliferative activity of EC-synthetic retinoids in Caco-2 cells [44]. Four sets of experiments were tested: first, Caco-2 cells were treated with serial dilutions of all retinoid compounds individually to detect their experimental IC_50_ values. Second, Caco-2 cells were treated with a combination of EC19 or EC23 with either CD437 or AC261066 agonist compounds in multiples or fractions of the IC_50_ (10, 5, 2, 1, 0.5 or 0.25× times the IC_50_), and the combined IC_50_ value was determined. Third, Caco-2 cells were treated with 1 µM CD2665 for 24 h in combination with the serial concentrations of ATRA or EC-retinoids. Fourth, Caco-2 cells were treated with 1 µM CD2665 for 8 h only, the culture media were washed out, and serial concentrations of ATRA or EC-retinoids were added for a further 24 h. The cellular viability was measured using a 3-(4,5-dimethylthiazol-2-yl)-2,5-diphenyltetrazolium bromide (MTT, Serva)) colorimetric assay [44]. The effectiveness of these combinations was measured by multi-drug effect analysis (isobologram) originally described by Chou and Talalay [143]. Briefly, the isobologram chart of each combination was created based on the IC_50_ concentration of each individual compound within the mixture, as previously described [38,132,133]. To calculate the combination index (CI), the following Equation (1) was used [144]:

CI = d1/D1 + d2/D2 (1)

in the previous equation, d1 and d2 are the doses of drug 1 and drug 2 that, when used in combination, produce a specific effect (here, a 50% reduction in cell viability). D1 and D2 are the doses of drug 1 and drug 2, that, when given individually, produce the same effect. Additionally, CI = 1, >1 and <1 indicate additivity, antagonism and synergy, respectively, [145]. The extent of synergism or antagonism was further refined by Chou [144].

To achieve a certain level of cytotoxicity, the fold reduction in the dose of each drug within mixtures, versus individual treatment, is referred to as the Dose Reduction Index (DRI). Here, this index was calculated from Equation (2) [146]. When the DRI is higher than one, the combination is considered effective, and thus a greater DRI value means a more favorable combination. However, a DRI equal to or lower than one indicates either no dose reduction or an unfavorable reduction, respectively; and in both cases, the combination is considered ineffective [147].

DRI (for drug 1) = D1/d1(2)

### 4.4. Flow Cytometry Analysis of the Combinatorial Effect of RAR-Agonists and EC-Synthetic Retinoids

Caco-2 cells were seeded into six-well plates at a density of 1 × 10^6^ cells and left for 24 h. After attachment, the cells were treated with the retinoids either individually or in combinations using the individual IC_50_ dose which was previously obtained from the isobologram analysis. Following 24 h, cell cycle and apoptosis assays were performed as previously mentioned [44]. Briefly, the cells were collected, fixed overnight and then stained with PI (50 µg/mL). The DNA content was measured by a Cytoflex flow cytometer (Beckman Coulter, IN, USA) and G0-G1, S, and G2M cells were gated as appropriate using CytExpert software (version 2.4.0.28).

### 4.5. Assessment of the Pro-Apoptotic and Pro-Necrotic Activity

The ability of EC19 and EC23 to induce Caspase-3/7-mediated apoptosis, in addition to necrosis, in Caco-2 cells was detected using a CellEvent™ Caspase-3/7 Green Flow Cytometry Assay Kit (Thermo Scientific, Waltham, MA, USA). The CellEvent™ Caspase-3/7 Green Detection Reagent consists of a four-amino acid peptide (DEVD) conjugated to a nucleic acid-binding dye. Thus, it is used as a fluorogenic substrate for detecting the activation of Caspases-3/7 enzymes. During apoptosis, active Caspase-3 and -7 stimulate DEVD peptide cleavage, liberating the DNA-binding dye with detectable fluorescence. In addition, the SYTOX™ AADvanced™ dead cell stain is provided with the kit (Thermo Scientific, Waltham, MA, USA) to easily discriminate apoptotic cells from live and necrotic cells. The manufacturer’s instructions were optimized and followed to perform this assay. In brief, 1 × 10^6^ Caco-2 cells were seeded in 25-cm^2^ flasks and allowed to adhere overnight at 37 °C. After reaching about 90% confluence, the cells were treated with or without the individual IC_50_ of EC-synthetic retinoids or ATRA, as we previously reported [44]. The cells were harvested with a rubber policeman and collected by centrifugation at 500× *g*. The cell pellet was resuspended in 1 mL of complete growth medium. Then, 1 µL of CellEvent™ Caspase-3/7 Green Detection Reagent was added to each sample, and the cell suspensions were incubated for 45 min at room temperature. During the final 5 min of incubation, 1 µL of the 1 mM SYTOX™ AADvanced™ Dead Cell Stain solution in DMSO was added to each sample. After 5 min, the samples were analyzed by a Cytoflex flow cytometer (Beckman Coulter, USA) without washing or fixation using excitation at 488 nm. The fluorescence emissions from the CellEvent™ Caspase-3/7 Green Detection Reagent and SYTOX™ AADvanced™ Dead Cell Stain were collected using 530/30 and 690/50 bandpass filters, respectively. Dual-parameter dot plots of CellEvent™ Caspase-3/7 Detection Reagent fluorescence versus SYTOX™ AADvanced™ fluorescence were created. The apoptotic and necrotic cell percentages were obtained by calculating the percentage ratio of apoptotic and necrotic cell events, respectively, to the whole cell population according to the standard protocol [51,52].

### 4.6. Measurement of Calcium-Independent ATPase Activity

The total activity of calcium-independent ATPase enzymes has been quantified colorimetrically as described by Chan and colleagues [148] after the exposure to individual IC_50_ doses of retinoids for 24 h. In this assay, the inorganic phosphate (Pi) liberated after the hydrolysis of ATP by ATPase enzymes was complexed into a colored product in one step, after the addition of the mixed reagent containing malachite green, ammonium molybdate and polyvinyl alcohol (2:1:1). The intensity of absorbance at 630 nm was measured using a BioTek 800 TS absorbance reader (BioTek, Winooski, VT, USA) and was directly proportional to the quantity of liberated Pi. The amount of Pi in each sample was determined using a standard curve prepared from solutions of potassium di-hydrogen phosphate (KH_2_PO_4_) with a known Pi concentration. Unlike other ATPase assay techniques, the removal of protein from the assay mixture was not necessary as it does not interfere with color formation [148,149].

Regarding the ATPase sample preparation, Caco-2 cells were seeded in 25 cm^2^ flasks at a density of 1 × 10^6^ and allowed to adhere overnight. The attached cells were then treated with the IC_50_ concentration of retinoids as mentioned in the previous report [44], followed by a 24 h incubation. All cellular ATPases were extracted from Caco-2 cells after lysis with 1% Triton X-100, 5 mM EDTA, 50 mM NaCl and 20 mM HEPES (pH 7.4). To normalize the total protein content in the sample, the concentration of all proteins in the sample was measured by a Bradford assay [150], and 200-µg total proteins were used per each ml of cell lysate [151]. For the assessment of the calcium-independent ATPase activity, the reactions were performed in the presence of 200 µM EDTA in addition to 1 mM ATP, 20 mM sodium azide and 50 mM Tris (pH 7.5) [148]. To account for the non-enzymatic hydrolysis of ATP, control reactions were made using only ATP in the reaction buffer and the absorbance intensity was then subtracted from the readings of samples and standards [152].

### 4.7. DNA Fragmentation Assay

DNA fragmentation is a hallmark of apoptosis. Although the TUNEL assay is widely used for detecting apoptotic cells, it has been shown to provide false positive signals in some necrotic cells [153,154,155]. The DNA fragmentation in Caco-2 cells was therefore quantified through the colorimetric diphenylamine assay as described [53,54,55]. Briefly, 1 × 10^6^ cells were seeded and left to attach overnight. Adhered cells were treated with or without the individual IC_50_ concentrations of ATRA, EC19 and EC23 for 24 h. After incubation, the cells were gently scraped and centrifuged at 300× *g* at 4 °C for 10 min to pellet the cells. The pelleted cells were resuspended in 0.8 mL of 0.01 M PBS (pH 7.4) and then lysed with 0.7 mL of ice-cold lysis buffer containing 0.5% Triton X-100, 20 mM EDTA and 5 mM Tris at pH 8.0. To ensure complete lysis, the mixture was incubated for 15 min at 4 °C. The cell lysate was centrifuged at 13,000× *g* at 4 °C. At this stage, fragmented DNA is present in the supernatant while intact DNA is in the pellets. The supernatant was transferred to a 5-mL glass tube, treated with 1.5 mL of 10% Trichloroacetic acid (TCA) and incubated for 10 min at room temperature. The pellet containing the non-fragmented DNA was resuspended in 1.5 mL of Tris-EDTA (TE) buffer containing 10 mM Tris and 1 mM EDTA at pH 8.0 followed by incubation with 1.5 mL of 10% TCA for 10 min at room temperature. Both the intact and fragmented DNA were collected from TCA suspensions by centrifugation at 500× *g* at 4 °C for 15 min. The pellet containing DNA was resuspended in 0.7 mL of 5% TCA, boiled at 100 °C to liberate the inorganic phosphate and then allowed to cool to room temperature. Next, the suspensions were centrifuged at 300× *g* at 4 °C and 0.5 mL of the resulting supernatant was transferred to a new glass tube. Finally, the color was developed after overnight incubation at 30 °C with a reagent containing 1.5 g diphenylamine dissolved in 100 mL acetic acid and 1.5 mL H_2_SO_4_ with acetaldehyde at a final concentration of 16 microgram/mL. After incubation, the intensity of absorbance was quantified colorimetrically at 600 nm in both the supernatant and pellet reflecting the amount of fragmented and intact DNA, respectively. The extent of DNA fragmentation was expressed as a relative ratio of low-molecular-weight, fragmented DNA to the total DNA content in the sample [55].

### 4.8. Immunocytochemistry Analysis of DNA Damage Using HCS DNA Damage

The Caco-2 cells were seeded onto coverslips at a density of 1 × 10^6^ cells using 0.1 mg/mL Poly-L-Lysine (Sigma-Aldrich, St. Louis, MO, USA) for immunocytochemistry (ICC) analysis. After treatment with the individual IC_50_ of EC-synthetic retinoids and ATRA, the cells were fixed in a formalin neutral buffer (FNB) solution. The fixed cells were permeabilized with 0.1% Triton-x 100 and then blocked with 1% bovine serum albumin (BSA, prepared in PBS containing 0.1% Tween 20) for 30 min. Then, the cells were incubated with the primary antibody of pH2AX antibody diluted with blocking buffer and incubated for 4 h. After incubation, the nuclei of cells were stained with Hoechst 33,342 diluted with blocking buffer (1:500). The coverslips were mounted in an anti-fading medium (0.1% p-phenylenediamine, dissolved in 90% glycerin/PBS) and the signals were detected using a Carl Zeiss LSM 710 confocal microscope (Carl Zeiss, Oberkochen, Germany).

### 4.9. Gene Expression Analysis Using RT-qPCR

The expression of genes coding for ABC transporters (P-gp1, BCRP and MRP1), apoptosis-linked ATPase (Hsp70) and copper efflux pump (ATP7A) were analyzed in Caco-2 cells after retinoids treatment using real-time quantitative PCR (RT-qPCR). In brief, 1 × 10^6^ cells were seeded in 25-cm^2^ flasks in triplets at the standard incubation conditions and allowed to adhere overnight. The attached cells were treated with or without the individual IC_50_ concentrations of retinoids and incubated for 24 h. After incubation, the cells were harvested, and the total RNA was extracted using a Favor-Prep^TM^ Blood/Cultured cell total RNA purification mini kit (Favorgen Biotech Corp., Ping-Tung, Taiwan). The purified RNA was then reverse transcribed into the first-strand cDNA using a Revert Aid First-Strand cDNA Synthesis Kit (Thermo Scientific, Waltham, MA, USA). All qPCR reactions were carried out using a HERA^PLUS^ SYBR^®^ Green qPCR Kit (Willowfort, Nottingham, UK). The differential gene expression was performed using the 2^–ΔΔCT^ method using β-actin as the reference gene [156]. The specificity of each gene amplification was verified at the end of the qPCR reactions through the melt curve analysis of the PCR products. The sequences of primers used are listed in Table 5.

### 4.10. Western Blotting Analysis

The expression of the MDR1, MRP1 and Hsp70 proteins in Caco-2 cells were determined using the Western blot procedure according to the protocol described earlier [38,148,149]. In brief, 1 × 10^6^ cells were seeded per well in six-well plates in triplets and then treated with an IC_50_ dose of ATRA, EC19 or EC23 for 24 h. After incubation, both the attached and floated cells were collected and lysed. The Caco-2 cell lysates were immediately collected, sonicated and centrifuged at 12,000× g for 15 min. The resulting supernatant was separated, and the total soluble protein concentration was determined colorimetrically using the Pierce™ 660 nm Assay (Thermo Scientific, Waltham, MA, USA). To ensure equal loading, 30 µg total protein from each sample was mixed with sodium dodecyl sulfate (SDS)-containing loading dye and then denatured by boiling for 5 min. After cooling on ice for 10 min, the samples were separated by SDS-polyacrylamide gel electrophoresis (Cleaver Scientific Ltd., UK). After electrophoresis, the protein bands were transferred onto PVDF membranes for 30 min using a Trans-Blot^®^ SD semi-dry transfer cell (Biorad, Hercules, CA, USA). The membranes were then blocked, washed and incubated with antibodies against MDR1 (1:1000, #12683; Cell Signaling Technology, Danvers, MA, USA), MRP1 (1: 1500, #72202, Cell Signaling Technology), Hsp70 (1:1000, #MA3-009, Thermo Scientific, Waltham, MA, USA) and β-actin (1:3000, #A5060, Sigma-Aldrich, St. Louis, MO, USA) for 16–17 h at 4 °C in a humidified chamber. The blots were washed and then incubated at room temperature with matched horseradish peroxidase (HRP)-linked secondary antibodies (Dako, Denmark) for 1.5 h. Finally, the bands were visualized with chemiluminescence Western Lightning ECL (Perkin Elmer, Waltham, MA) for 1 min in a Chemi-Doc imager (Biorad, Hercules, CA, USA) and analyzed with Bio-Rad Image Lab software with normalization to β-actin (original Western blot is shown in Supplementary File S1).

## 5. Conclusions

The present study illustrated the potential molecular mechanisms mediated by two synthetic retinoids called EC19 and EC23 using a group of molecular assays and the Caco-2 cancer cell line as sensitive models for colorectal carcinoma. The in vitro approach revealed that EC-synthetic retinoids, especially EC19, hold promise as an effective strategy for the treatment of cancer-based on their significant effects in reducing ATPase activity and ABC transporters and inducing DNA fragmentation, apoptosis and necrosis. The study suggested that combinations of EC-synthetic retinoids with CD437 and AC261066 as selective RAR-agonists present synergistic anti-cancer strategy. The combinatorial effects of retinoids include lowering their IC_50_ doses, the enhancement of early and late apoptotic effects, the induction of cell cycle arrest at G0-G1 in addition to G2M phase arrest. Some key regulatory genes and proteins were modulated, such as *ATP7A*, *ABCG2*, *ABCC1* and *ABCB1* in response to EC-synthetic retinoids, suggesting that their expression may be under the regulation of retinoid signaling pathways with a potential role in diminishing cancer resistance and carcinogenesis. Indeed, the current data will help in continuing the molecular investigations and beyond using the promising EC-synthetic retinoids for their potential use in in vivo and clinical use and for the future optimization of novel retinoid-based therapies that might be helpful for solid tumor patients.

## Figures and Tables

**Table 1:** The in vitro antiproliferative activity and Isobologram analysis of the individual retinoids and combinations with CD437, AC261066 and CD2665 (using fixed ratios of the individual IC_50_ values) in Caco-2 cells. Dose reduction index (DRI) and combination index (CI) was calculated. Data represent mean ± SEM, *n* = 3.

Drug(s) (Single/Combinations)	^#^ IC_50_ (µM) ± SEM (Single)	^#^ IC_50_ (µM) ± SEM (Combination)	×IC_50_ (µM) (Combination)	Individual * IC_50_ (µM) (within the Combination)		DRI	CI
				Retinoid (A)	Retinoid (B)		
Individual Retinoids							
ATRA	97.70 ± 9.0	-----	-----	-----	-----	-----	-----
EC19	27.20 ± 1.8	-----	-----	-----	-----	-----	-----
EC23	23.00 ± 1.2	-----	-----	-----	-----	-----	-----
CD437	2.80 ± 0.7	-----	-----	-----	-----	-----	-----
AC261066	26.90 ± 2.1	-----	-----	-----	-----	-----	-----
CD2665	27.90 ± 3.1	-----	-----	-----	-----	-----	-----
Agonists combinations							
ATRA (A) and CD437 (B)	-----	5.8 ± 1.2 ^ns^	2.07	202.23	5.80	0.48	4.1
EC19 (A) and CD437 (B)	-----	<0.1 ***	0.04	1.09	0.11	24.95	0.1
EC23 (A) and CD437 (B)	-----	0.82 ± 0.1 **	0.29	6.67	0.81	3.45	0.6
ATRA (A) and AC261066 (B)	-----	47.90 ± 7.0 ^ns^	1.78	173.9	47.89	0.56	3.6
EC19 (A) and AC261066 (B)	-----	1.37 ± 0.3 ***	0.05	4.89	1.34	5.56	0.2
EC23 (A) and AC261066 (B)	-----	1.76 ± 0.4 ***	0.07	6.84	1.88	3.36	0.4
Antagonist combination							
CD2665 (A) and ATRA (B)	-----	1.70 ± 0.3 ***	0.06	1.67	5.86	16.67	0.12
CD2665 (A) and EC19 (B)	-----	24.28 ± 2.5 ^ns^	0.87	24.27	23.66	1.14	1.74
CD2665 (A) and EC23 (B)	-----	25.80 ± 4.2 ^ns^	0.92	25.67	21.16	1.1	1.84
CD2665 (A) then ATRA (B)	-----	61.4 ± 6.6 ^ns^	2.201	61.41	215.04	0.45	8.3
CD2665 (A) then EC19 (B)	-----	2.97 ± 0.8 ***	0.106	2.96	2.88	9.44	0.2
CD2665 (A) then EC23 (B)	-----	4.27 ± 0.3 ***	0.153	4.27	3.52	6.53	0.3

^#^ IC_50_ values were determined by non-linear regression of dose-response using the 4-parameter logistic model (4PL) in GraphPad prism 7. The statistical significance has been inferred by comparing the combined IC_50_ of the mixture to the individual single IC_50_ of the agonist/antagonist. * *p* < 0.05, ** *p* < 0.01, *** *p* < 0.001. ^ns^; non-significant (*p* > 0.05). ×IC_50_ represents the amplitude of change relative to the IC_50_ concentration of the agonistic retinoids.

**Table 2:** The percentage of viable, apoptotic, late apoptotic and necrotic cells was measured by AV/PI assay using flow cytometry. The assay was performed after the treatment of Caco-2 cells for 24 h with ATRA, EC19, EC23, CD437, AC261066 and combinations with either CD437 or AC261066 compared to 0.1% DMSO negative control. Data represent mean ± standard error of the mean (SEM), *n* = 3.

Retinoids (Individual and Combinations)	Apoptosis Analysis of Caco-2 Cell Line ^#^			
	% Viable Cells (LL)	% Early Apoptotic Cells (UL)	% Late Apoptotic Cells (LR)	% Necrotic Cells (UR)
Control	98.11 ± 5.6	0.00	1.87 ± 0.6	0.02
ATRA	67.21 ± 4.5 *	27.40 ± 1.6 ***	1.27 ± 0.6	4.12 ± 0.7 ***
EC19	64.80 ± 3.5 *	11.04 ± 2.1 ***	5.06 ± 1.2 *	19.10 ± 2.0 ***
EC23	76.00 ± 4.8 *	15.76 ± 2.5 ***	1.77 ± 0.9	6.47 ± 1.3 ***
CD437	62.23± 5.2 *	17.42 ± 1.4 ***	7.51 ± 0.46 **	12.84 ± 1.1 ***
AC261066	67.21 ± 4.7 *	27.40 ± 2.8 ***	1.27 ± 0.91	4.12 ± 1.9 ***
ATRA + CD437	39.86 ± 2.5 **	43.33 ± 3.6 ***	3.44 ± 1.0 *	13.37 ± 1.3 ***
EC19 + CD437	36.68 ± 1.9 **	48.15 ± 4.1 ***	3.41 ± 1.1 *	11.76 ± 4.1 ***
EC23 + CD437	41.17 ± 2.2 **	41.57 ± 2.9 ***	4.33 ± 1.4 *	12.93 ± 2.2 ***
ATRA+ AC261066	31.59 ± 1.5 **	55.39 ± 3.5 ***	2.38 ± 1.1 *	10.64 ± 1.6 ***
EC19 + AC261066	20.01 ± 2.1 ***	63.43 ± 5.3 ***	0.91 ± 0.3	15.65 ± 2.5 ***
EC23 + AC261066	33.91 ± 1.6 **	52.21 ± 5.2 ***	3.29 ± 1.3 *	10.59 ± 1.7 ***

* *p* < 0.05, ** *p* < 0.01 and *** *p* < 0.001. *p* values indicate (either increase or decrease) the significance in comparison to untreated control cells (0.1%DMSO solvent only). ^#^ LL, lower lift; UL, upper lift; LR, lower right; UR, upper right quadrants.

**Table 3:** Cell cycle analysis of Caco-2 cells after ATRA, EC19 and EC23 and agonists treatment for 24 h and in combinations with either CD437 or AC261066 compared to 0.1% DMSO negative control showing the DNA content at different cycle phases.

Retinoids (Individual and Combinations)	Cell Cycle Analysis of Caco-2 Cell Line ^#^			
	% SubG_0_-G_1_	% G_0_-G_1_	% S	% G_2_M
Control	0.09	53.46 ± 3.9	13.01 ± 1.3	33.07 ± 2.2
ATRA	0.00	0.67 ***	4.25 ± 3.2 ***	94.73 ± 5.4 ***
EC19	0.00	8.32 ± 1.4 ***	20.15 ± 2.2 *	70.86 ± 6.1 ***
EC23	0.00	3.28 ± 0.9 ***	7.58 ± 3.9 *	88.86 ± 5.4 ***
CD437	0.00	3.78 ± 0.9 ***	8.76 ± 0.9 *	86.99 ± 0.9 ***
AC261066	0.00	2.20 ± 0.9 ***	7.49 ± 0.9 *	90.16 ± 0.9 ***
ATRA + CD437	11.75 ± 4.8 ***	42.08 ± 4.2 *	20.17 ± 3.5 *	25.54 ± 3.7
EC19 + CD437	22.54 ± 5.2 ***	36.06 ± 2.1 *	25.70 ± 2.4 *	15.51 ± 2.9 *
EC23 + CD437	23.76 ± 3.4 ***	51.60 ± 4.3	10.23 ± 1.7	14.34 ± 2.2 *
ATRA+ AC261066	18.63 ± 2.1 ***	38.47 ± 2.2 *	23.53 ± 2.9 *	19.01 ± 1.9 *
EC19 + AC261066	52.24 ± 3.6 ***	30.83 ± 1.6 *	12.34 ± 3.1	4.63 ± 2.8 ***
EC23 + AC261066	36.02 ± 2.5 ***	41.07 ± 2.9 *	10.93 ± 1.1	11.99 ± 7.1 *

^#^ Data represent mean ± SEM, *n* = 3. The significance in p-values for comparison with control untreated cells (0.1%DMSO solvent only) is indicated (either increase or decrease) as * *p* < 0.05, *** *p* < 0.001.

**Table 4:** Distribution of Caco-2 cell populations after exposure to individual retinoids as detected by CellEvent™ Caspase-3/7 Green- SYTOX™ Flow Cytometry Assay Kit.

Key Parameters	Control ^#^	ATRA ^#^	EC19 ^#^	EC23 ^#^
% Live cells (SYTOX™ −ve/Caspase-3/7 −ve)	80.56 ± 6.39	4.66 ± 3.35 **	12.31 ± 1.09 **	4.69 ± 0.33 **
% Apoptotic cells (SYTOX™ −ve/Caspase-3/7 +ve)	1.74 ± 1.22	11.75 ± 2.92 *	6.70 ± 1.36 *	11.76 ± 0.88 *
% Necrotic cells (SYTOX™ +ve/Caspase-3/7 +ve)	10.75 ± 1.69	82.69 ± 7.13 *	79.05 ± 1.384 *	81.75 ± 3.83 *

^#^ Data are represented as mean ± SEM of three independent experiments. * *p* < 0.05 and ** *p* < 0.01 indicate significant difference from control.

**Table 5:** Sequences of primers used in reverse-transcriptase polymerase chain reactions (RT-qPCR).

Gene	Primer Sequence
P-glycoprotein 1/*ABCB1*	F: 5′-TCACCAAGCGGCTCCGATACAT-3′ R: 5′-CCCGGCTGTTGTCTCCATAGGC-3′
BCRP/*ABCG2*	F: 5′-TATAGCTCAGATCATTGTCACAGTC-3′ R: 5′-GTTGGTCGTCAGGAAGAAGAG-3′
MRP1/*ABCC1*	F: 5′-CGGAAACCATCCACGACCCTAATC -3′ R: 5′-ACCTCCTCATTCGCATCCACCTGG-3′
Hsp70	F: 5′-ACACGAATCCCTGCGGTAAAA-3′ R: 5′-GCAGGCGATAAGATGGCACA-3′
ATP7A	F: 5′-TGAACAGTCATCACCTTCATCGTC-3′ R: 5′-GCGATCAAGCCACACAGTTCA-3′
β-actin	F: 5′-GCACCACACCTTCTACAATGAGC-3′ R: 5′-GGATAGCACAGCCTGGATAGCAAC-3′

## Data Availability

The data presented in this study are available in this article.

## Supplementary Materials

The supporting information can be downloaded at: https://www.mdpi.com/article/10.3390/ijms23169442/s1.
