# Supplementary material for: Augmented Therapeutic Potential of EC-Synthetic Retinoids in Caco-2 Cancer Cells Using an In Vitro Approach
Source: Int J Mol Sci. 2022 Aug 21;23(16):9442. doi: 10.3390/ijms23169442 (PMC9409216; doi:10.3390/ijms23169442)
Supplement: Supplementary file 1 [file ijms-23-09442-s001.zip › ijms-1823355-supplementary.pdf]

Supplementary figures for
“Augmented therapeutic potential of EC-synthetic retinoids in Caco-2 cancer cells using *in-vitro* approach”

Authored by **Mohamed R. Abdelaal, Esra[illegible] Ibrahim, Mohamed R. Elnagar, Sameh H[illegible] Soror, and Hesham Haffez**

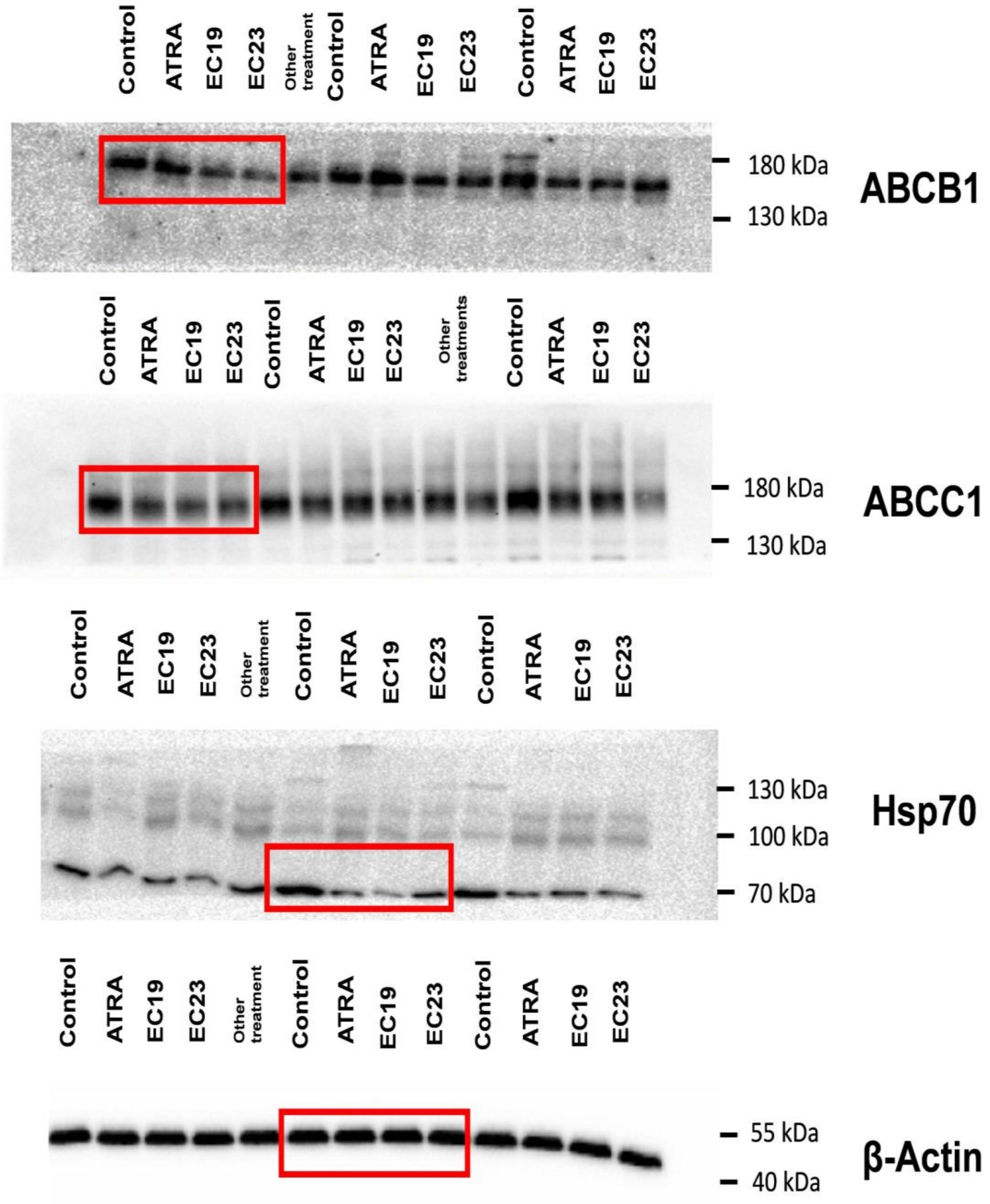

S1. Supplementary figure for the original source of Western blot images showed in **Fig.6** illustrating effect of IC_{50} dose of ATRA, EC19, and EC23 on the expression levels of ABCB1 (MDR1), ABCC1 (MRP1), Hsp70 and β-Actin proteins in Caco-2 cells.
